# Novel insights into the crosstalk between non-coding RNA and apoptosis in rheumatoid arthritis: diagnostic functions and therapeutic applications

**DOI:** 10.3389/fimmu.2025.1550839

**Published:** 2025-05-16

**Authors:** Jianting Wen, Jian Liu, Lei Wan, Fanfan Wang

**Affiliations:** ^1^ Department of Rheumatology and Immunology, First Affiliated Hospital of Anhui University of Chinese Medicine, Hefei, Anhui, China; ^2^ Institute of Rheumatology, Anhui Academy of Chinese Medicine, Hefei, Anhui, China; ^3^ Anhui Province Key Laboratory of Modern Chinese Medicine, Department of Internal Medicine Application Foundation Research and Development, Hefei, Anhui, China

**Keywords:** ncRNAs, rheumatoid arthritis, apoptosis, therapeutic applications, TCM

## Abstract

Rheumatoid arthritis (RA) is a chronic autoimmune inflammatory disorder and a leading cause of disability worldwide, significantly impairing patients’ quality of life. As current therapeutic options remain limited, there is an urgent need for novel strategies, including the use of medicinal plants, to delay the development and progression of RA. Acute inflammation in RA is often accompanied by impaired apoptosis, which contributes to disease pathogenesis. With advances in high-throughput sequencing technologies, an increasing number of non-coding RNAs (ncRNAs) have been identified and extensively studied for their roles in both physiological and pathological processes. Dysregulation of these ncRNAs—particularly long non-coding RNAs (lncRNAs) and circular RNAs (circRNAs)—has been implicated in various disorders, including RA. Given the well-established association between apoptosis and ncRNA expression in RA, a comprehensive understanding of their intricate interplay is essential. In this study, we systematically explore the complex interactions between lncRNAs and circRNAs in regulating apoptosis during the pathogenesis of RA. Additionally, we highlight emerging evidence, suggesting that ncRNA-mediated modulation of apoptosis can be achieved through herbal medicines, offering promising therapeutic avenues for RA treatment.

## 1 Introduction

Rheumatoid arthritis (RA) is a chronic autoimmune inflammatory disease, and its pathogenesis is strongly associated with immune system dysregulation (1). In addition to joint involvement, RA often presents with extra-articular manifestations that can affect multiple organs and systems, including the lungs, heart, and hematological system (2, 3). Prolonged joint pain, swelling, deformity, functional impairment, and the long-term use of medications not only increase the financial burden on patients but also significantly diminish the quality of life and interfere with normal joint function (4). Fibroblast-like synoviocytes (FLSs) are key contributors to the pathogenesis of RA. They play a central role in the aberrant remodeling of the synovial membrane and drive the perpetuation of inflammation and joint damage (5, 6).

Apoptosis, a form of programmed cellular death, is critically involved in the development of RA, contributing to various pathological mechanisms (7). Morphologically, apoptosis is characterized by cell shrinkage, DNA fragmentation, chromatin condensation, and the formation of apoptotic bodies that package cellular debris. The efficient clearance of these apoptotic bodies is essential to prevent secondary inflammation. In RA, insufficient apoptosis and abnormal proliferation of FLS have been well documented. The accumulation of hyperplastic FLS contributes significantly to the various pathological processes of RA (8). In addition, macrophages in the RA synovium often evade apoptosis, thereby sustaining the inflammatory milieu and exacerbating joint damage. Therefore, strategies that promote macrophage apoptosis within RA-affected joints hold promise for advanced therapeutic interventions (9). Furthermore, in RA cartilage, chondrocyte apoptosis—mediated by p53 activation and downregulation of Bcl-2—has been linked to the severity of cartilage degradation (10).

Non-coding RNAs (ncRNAs) are RNA molecules transcribed from genes that do not encode proteins, yet they constitute over 90% of human gene transcripts and play crucial roles in both physiological processes and the pathogenesis of complex diseases (11, 12). According to their length, ncRNAs are broadly categorized into small ncRNA (<200 nt), such as microRNAs (miRNAs), and long non-coding RNA (lncRNA; >200 nt) (13, 14). Additionally, circular RNAs (circRNAs) are a unique class of ncRNAs characterized by a covalently closed loop structure, which confers enhanced stability compared to linear RNAs (15, 16). lncRNAs and circRNAs are known to modulate the biological behaviors of RA-FLS through Competitive endogenous RNA (ceRNA) mechanisms. In this context, lncRNAs and circRNAs can act as “miRNA sponges”—competing with target messenger RNAs (mRNAs) for miRNA binding and thereby indirectly regulating gene expression by sequestering miRNAs away from their target transcripts (17, 18).

At present, there is no clinically curative treatment for RA, and many commonly used drugs are associated with adverse side effects. Traditional Chinese medicine (TCM) is rooted in the principles of syndrome differentiation and a holistic approach (19). For instance, *Tripterygium wilfordii* and its derivative formulations have been extensively used for the clinical treatment and prevention of RA. One study identified a novel ceRNA axis (lncRNA ENST00000494760/miR-654-5p/C1QC) as a potential biomarker for the therapeutic effects of Tripterysium Glycosides Tablets in RA (20). Similarly, Xinfeng capsule (XFC) has been shown to alleviate inflammation and hypercoagulability in RA by regulating the lncRNA down syndrome critical region 9 (DSCR9)/ribosomal protein lateral stalk subunit P2 (RPLP2)/phosphatidylinositol 3-kinase (PI3K)/protein kinase B, PKB (AKT) axis (21). TCM exhibits the advantages of acting through multiple targets, pathways, and mechanisms, offering effective treatment outcomes with fewer side effects (22).

In this study, we first discuss the role of apoptosis in RA pathogenesis, followed by an analysis of how lncRNAs and circRNAs, functioning as ceRNA, influence apoptosis in RA. Finally, we explore how targeting the ceRNAs/apoptosis axis may provide promising therapeutic strategies for the treatment of RA.

## 2 lncRNA/circRNA involved in the pathogenesis of RA

### 2.1 lncRNAs/circRNAs as diagnostic markers for RA

**Figure 1:**
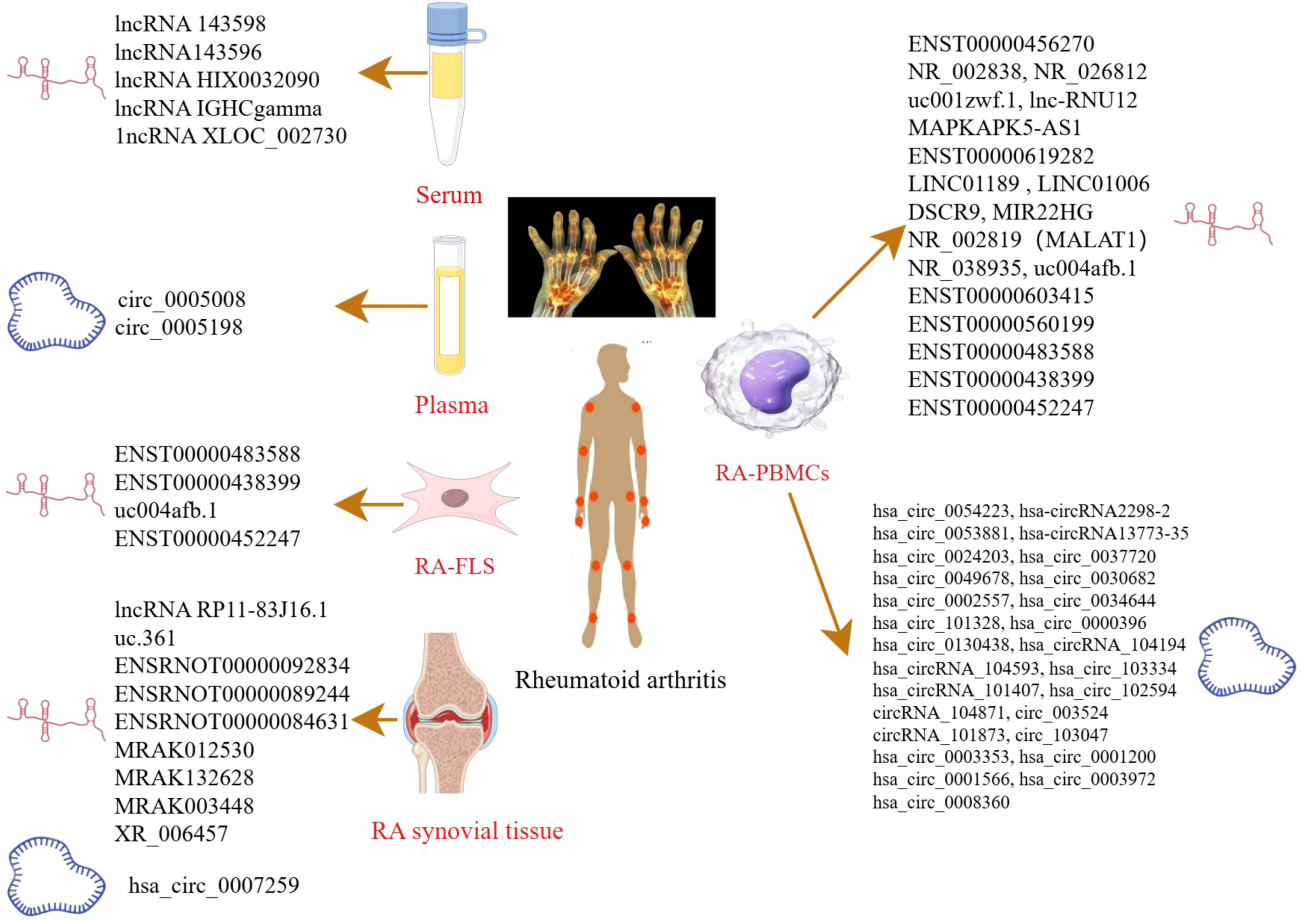
LncRNAs/circRNAs as RA diagnostic markers (by Figdraw). This figure summarizes the lncRNAs and circRNAs identified in distinct biological samples derived from RA patients, including serum, plasma, peripheral blood mononuclear cell (PBMCs), FLS, and synovial tissues.

Recent studies have demonstrated that lncRNAs and circRNAs can regulate gene expression at multiple levels, including epigenetic, transcriptional, and post-transcriptional mechanisms. Through these regulatory pathways, they participate in diverse biological processes, making them promising biomarkers for the diagnosis of numerous diseases (23). Notably, aberrant expression of lncRNAs and circRNAs has been strongly related to RA, suggesting their potential utility as diagnostic markers or therapeutic targets. Detailed findings are provided in **Table 1**, **Figure 1**.

**Table 1:** lncRNAs/circRNAs as RA diagnostic markers.

Specimens	Methods	Differentially expressed ncRNAs	Roles	Expression level	References
Serum	Microarray	lncRNA 143598, lncRNA143596, lncRNA HIX0032090, lncRNA IGHCgamma1, and lncRNA XLOC_002730	Involved in signaling pathways of TLRs, NF-κB, and IRF3/IRF7-mediated signaling transduction	Upregulation	(24)
Plasma	Microarray	289 DE lncRNAs	Involved in the pathogenesis of RA mainly through platelets	169 upregulated and 120 downregulated	(25)
PBMCs	Microarray analysis	ENST00000456270, NR_002838, NR_026812, and uc001zwf.1	Linked to the disease activity of RA	ENST00000456270 and NR_002838 were upregulated. NR_026812 and uc001zwf.1 were downregulated.	(26)
PBMCs	Genome-wide lncRNA microarray screening	Lnc-RNU12	Influenced the T cell cycle by targeting c-JUN and CCNL2	Downregulation	(27)
PBMCs	RNA sequencing	MAPKAPK5-AS1, ENST00000619282, LINC01189, LINC01006, DSCR9, and MIR22HG	Related to apoptosis and autophagy	MAPKAPK5-AS1, LINC01189, and DSCR9 were upregulated ENST00000619282, LINC01006, and MIR22HG were downregulated	(28)
PBMCs	Microarray analysis	NR_002819 (MALAT1), NR_038935, ENST00000603415, and ENST00000560199	Diagnosis of RA-ILD	NR_002819 (MALAT1), NR_038935, and ENST00000603415 were upregulated. ENST00000560199 was downregulated.	(29)
RA-FLS	Microarray analysis	ENST00000483588, ENST00000438399, uc004afb.1, and ENST00000452247	Potential value for the diagnosis and assessment of the disease activity of RA	ENST00000483588 was upregulated. ENST00000438399, uc004afb.1, and ENST00000452247 were downregulated.	(30)
Synovial tissues	RNA sequencing	lncRNA RP11-83J16.1	Promoted RA-FLS proliferation, migration, invasion, and inflammation by regulating URI1 and downstream β-catenin pathway components	ENST00000438399, uc004afb.1, and ENST00000452247 were downregulated.	(31)
Synovial tissues	Epitranscriptomic microarray	Hsa_circ_0007259	Regulated inflammation through miR-21-5p/STAT3	Upregulation	(40)
PBMCs	RNA sequencing	Hsa_circ_0054223, hsa-circRNA2298-2, hsa_circ_0053881, hsa-circRNA13773-35, hsa_circ_0024203, hsa_circ_0037720, hsa_circ_0049678, hsa_circ_0030682, hsa_circ_0002557, and hsa_circ_0034644	Related to cellular protein metabolic processes, gene expression, the immune system	Hsa_circ_0054223, hsa-circRNA2298-2, hsa_circ_0053881, hsa-circRNA13773-35, and hsa_circ_0024203 were upregulated. hsa_circ_0037720, hsa_circ_0049678, hsa_circ_0030682, hsa_circ_0002557, and hsa_circ_0034644 were downregulated.	(32)
PBMCs	Gene microarray technology	Hsa_circ_101328	Associated with C-reactive protein (CRP)	Upregulation	(33)
Plasma	Microarray	circ_0005008 and circ_0005198	Linked to the disease activity of RA	Upregulation	(34)
PBMCs	Microarray	Hsa_circ_0000396 and hsa_circ_0130438	Associated with inflammation and transcriptional activity	Downregulation	(35)
PBMCs	Microarray	Hsa_circRNA_104194, hsa_circRNA_104593, hsa_circRNA_103334, hsa_circRNA_101407, and hsa_circRNA_102594	Influence the occurrence and development of RA	hsa_circRNA_104194 was upregulated. hsa_circRNA_104593, hsa_circRNA_103334, hsa_circRNA_101407, and hsa_circRNA_102594 were downregulated.	(36)
PBMCs	Microarray	circRNA_104871, circRNA_003524, circRNA_101873, and circRNA_103047	AS potential biomarkers for RA patient diagnosis	Upregulation	(37)
PBMCs	RNA sequencing	Hsa_circ_0003353	Play an essential role in promoting immunity, inflammation, synovial invasion, and joint destruction	Upregulation	(39)
PBMCs	RNA sequencing	Hsa_circ_0001200, hsa_circ_0001566, hsa_circ_0003972, and hsa_circ_0008360	Influence the occurrence and development of RA	Hsa_circ_0001200, hsa_circ_0001566, and hsa_circ_0003972 were upregulated.	(38)
synovial tissue CIA	lncRNA-mRNA chip technology	uc.361, ENSRNOT00000092834, ENSRNOT00000089244, and ENSRNOT00000084631	Bi Zhong Xiao decoction reversing bone erosion in CIA rats from the perspective of lncRNA and mRNA	Upregulation	(41)
RA-FLS	genome-wide microarray assay	394 genes	(5R)-5-hydroxytriptolide influences the FLS cells systemically and especially in the process of immune-related pathways through lncRNAs.	281 downregulated and 113 upregulated	(42)
Arraystar rat	lncRNA/mRNA microarray	MRAK012530, MRAK132628, MRAK003448, and XR_006457	lncRNAs could be selected as critical therapeutic targets of astragalosides.	MRAK012530, MRAK132628 were upregulated. MRAK003448 and XR_006457 were downregulated	(43)

Xu et al. identified five significantly upregulated lncRNAs (RNA143598, RNA143596, HIX0032090, IGHCgamma1, and XLOC_002730) in the serum of RA patients using microarray analysis. These lncRNAs were intimately linked to IRF3/IRF7-mediated signal transduction pathways (24). Similarly, Qin et al. profiled plasma lncRNA expression in RA patients for the first time, identifying 169 upregulated and 120 downregulated lncRNAs. Additional lncRNAs were subsequently identified in PBMCs using RNA sequencing (RNA-seq) (25). Another microarray-based study of PBMCs in RA patients revealed five differentially expressed lncRNAs: two were downregulated (NR_026812 and uc001zwf.1), and two were upregulated (ENST00000456270 and NR_002838) (26). Moreover, a novel lncRNA, lnc-RNU12, was found to be downregulated in PBMCs and T-cell subtypes of RA patients. This lncRNA appears to influence the T-cell cycle by targeting cellular Jun proto-oncogene (c-JUN) and Cyclin L2 (CCNL2) (27). These findings underscore the critical roles of newly discovered lncRNAs in RA pathogenesis and open new avenues for mechanistic insights and diagnostic applications. A recent RNA-seq study identified seven apoptosis- and autophagy-related lncRNAs (MAPKAPK5-AS1, ENST00000619282, C5orf17, LINC01189, LINC01006, DSCR9, and MIR22HG), all of which were significantly correlated with clinical indices in RA (28). Interstitial lung disease (ILD), a common extra-articular manifestation of RA, has also been linked to specific lncRNA signatures. In a study focused on middle-aged female RA-ILD patients, three lncRNAs (NR_002819, NR_038935, and ENST00000603415) were upregulated, whereas ENST00000560199 was downregulated. These four lncRNAs may serve as potential biomarkers for the diagnosis and evaluation of RA-ILD in this patient subgroup (29). Identification of these lncRNAs could contribute to novel strategies for the prevention and treatment of RA. Zhang et al. reported 135 differentially expressed lncRNAs in RA-FLSs compared with normal FLSs. Among these, ENST00000483588 was upregulated, whereas ENST00000438399, uc004afb.1, and ENST00000452247 were downregulated, suggesting their involvement in RA pathology (30). In another study, Piao et al. identified 321 dysregulated lncRNAs in synovial tissues from RA patients versus healthy individuals (31). The expression levels of lncRNAs such as MTCO2P12, KCNQ5-IT1, and RP11-83J16.1 were significantly elevated, whereas LINC00570, RP11-342M1.6, and REXO1L4P were markedly decreased. Among them, lncRNA RP11-83J16.1 showed a positive correlation with inflammatory markers and disease activity. Functionally, it promoted RA-FLS proliferation, migration, invasion, and inflammation of RA-FLSs by regulating URI1 and activating the downstream β-catenin pathway. Therefore, lncRNA RP11-83J16.1 may represent a novel therapeutic target, offering deeper insights into RA pathogenesis. Elucidating the mechanism by which lncRNA regulates apoptotic pathways in RA could enhance predictive accuracy for therapeutic outcomes and guide the development of more effective treatment strategies.

Several studies have shed light on the critical roles of circRNAs in RA development. Using microarray analysis, researchers identified 35,342 differentially expressed circular RNAs (DEcircRNAs) in RA patients compared to healthy controls and 6,146 DEcircRNAs when compared to osteoarthritis (OA) patients (32). Further pathway analysis revealed that these DEcircRNAs were closely related to immune system regulation and the PI3K-Akt signaling pathway. Such circRNAs may serve as useful indicators for evaluating RA disease activity. In another study, Lu et al. identified hsa_circ_101328 as a promising biomarker for the early diagnosis of RA by gene microarray technology (33). Similarly, a microarray analysis of RA plasma samples revealed that circ_0005008 and circ_0005198 were significantly elevated and positively correlated with the severity of the disease (34). RNA-seq of PBMCs has also uncovered multiple circRNAs differentially expressed in RA. For instance, hsa_circ_0000396 and hsa_circ_0130438 were downregulated in RA patients, and receiver operating characteristic (ROC) curve analysis showed that these two circRNAs possess considerable diagnostic value for RA (35). Expression patterns of hsa_circRNA_104194, hsa_circRNA_104593, hsa_circRNA_103334, hsa_circRNA_101407, and hsa_circRNA_102594 were consistent with microarray data, suggesting their involvement in RA pathogenesis (36). Additionally, circRNA_104871, circRNA_003524, circRNA_101873, and circRNA_103047 were found to be significantly elevated in PBMCs from RA patients, indicating their potential as diagnostic biomarkers (37). Expression levels of hsa_circ_0001200, hsa_circ_0001566, hsa_circ_0003972, and hsa_circ_0008360 also mirrored findings from RNA-seq data, further supporting their association with RA progression (38). These findings offer valuable insights into the diagnostic and therapeutic potential of lncRNAs in RA. In another RNA-seq study, hsa_circ_0003353, a circRNA linked to immune and inflammatory processes, was found to be upregulated in RA patients. It appears to promote inflammation, synovial invasion, and joint destruction (39). Elevated levels of this circRNA in clinical blood samples were positively correlated with disease activity, underscoring its clinical significance. More recently, hsa_circ_0007259 was identified as hypermethylated and capable of competitively binding hsa_miR-21-5p, thereby promoting STAT3 expression. These findings suggest that hsa_circ_0007259 may serve as both a potential biomarker and a therapeutic target in RA diagnosis (40).

Collectively, these findings underscore the therapeutic potential of ncRNAs in RA, including in the context of TCM. For example, He et al. investigated the lncRNA expression profile in collagen-induced arthritis (CIA) rats treated with Bi Zhong Xiao Decoction. They identified four lncRNAs (uc.361-, ENSRNOT00000092834, ENSRNOT00000089244, and ENSRNOT00000084631) that may serve as therapeutic targets, primarily involved in immune-related biological processes (41). A previous study investigated the effect of astragaloside IV (AST) in adjuvant arthritis (AA) rats and identified four lncRNAs (MRAK012530, MRAK132628, MRAK003448, and XR_006457) as critical therapeutic targets of AST (42), highlighting its potential in RA treatment. Further, genome-wide microarray analysis in RA-FLS treated with (5R)-5-hydroxytriptolide (LLDT-8) revealed 394 differentially expressed lncRNAs (281 downregulated and 113 upregulated). These lncRNAs were primarily involved in immune regulation, suggesting that LLDT-8 may exert therapeutic effects through modulation of lncRNA expression (43). These ncRNAs not only offer novel insights into disease mechanisms but also hold great promise as biomarkers and therapeutic targets.

### 2.2 lncRNAs/circRNAs functioning as ceRNA in the pathogenesis of RA

miRNAs are key regulators of gene expression, primarily mediating their effects through mRNA degradation or translational repression. Emerging evidence has demonstrated that ncRNAs can function as ceRNA. Through binding to shared miRNA response elements, these ncRNAs act as molecular “sponges” that sequester miRNAs, thereby preventing them from repressing their target mRNAs. This ceRNA regulatory network has emerged as a significant mechanism underlying the pathogenesis of various diseases (44).

Recent studies have revealed the involvement of several lncRNA-mediated ceRNA axes in RA. For instance, lncRNA NR-133666 was shown to promote the proliferation and migration of RA-FLS by regulating the miR-133c/MAPK1 axis (45). In another study, lncRNA HIX003209 facilitated inflammatory responses in RA by sponging miR-6089, thereby activating the toll-like receptor 4 (TLR4)/nuclear factor kappa-light-chain-enhancer of activated B cells (NF-κB) signaling pathway (46). These findings not only expand our understanding of lncRNA function in RA but also suggest that these ceRNA axes could serve as novel therapeutic targets. Similarly, several circRNAs have been implicated in RA via ceRNA mechanisms. For example, circRNA_09505 was found to exacerbate inflammation and joint destruction in CIA mice through the miR-6089/AKT1/NF-κB axis (47). Furthermore, recent evidence highlights the role of m6A modifications in modulating circRNA activity. Specifically, circ_0066715, an m6A-modified circRNA, was shown to participate in RA pathogenesis by sponging miR-486-5p and subsequently enhancing ETS1 expression (48). Mechanistically, circ_0066715 inhibits the suppressive effect of miR-486-5p on ETS1, thereby promoting ETS1 expression. The circ_0066715/miR-486-5p/ETS1 axis represents a potential therapeutic target for RA intervention.

In conclusion, the involvement of lncRNAs and circRNAs in the pathogenesis of RA through ceRNA mechanisms provides novel insights into disease progression and potential therapeutic intervention. A deeper understanding of these regulatory networks could pave the way for innovative diagnostic tools and targeted therapies, ultimately improving clinical outcomes for RA patients.

## 3 The mechanisms of apoptosis in RA

### 3.1 Process of apoptosis

Apoptosis, a form of programmed cell death, is a well-characterized and extensively studied cellular process that plays a vital role in maintaining tissue homeostasis (49). Traditionally regarded as a passive and terminal fate of damaged cells, apoptosis is now recognized as an active, tightly regulated biological mechanism that serves as a fundamental component of immune defense, particularly in response to cellular damage from toxic or harmful stimuli (50).

**Figure 2:**
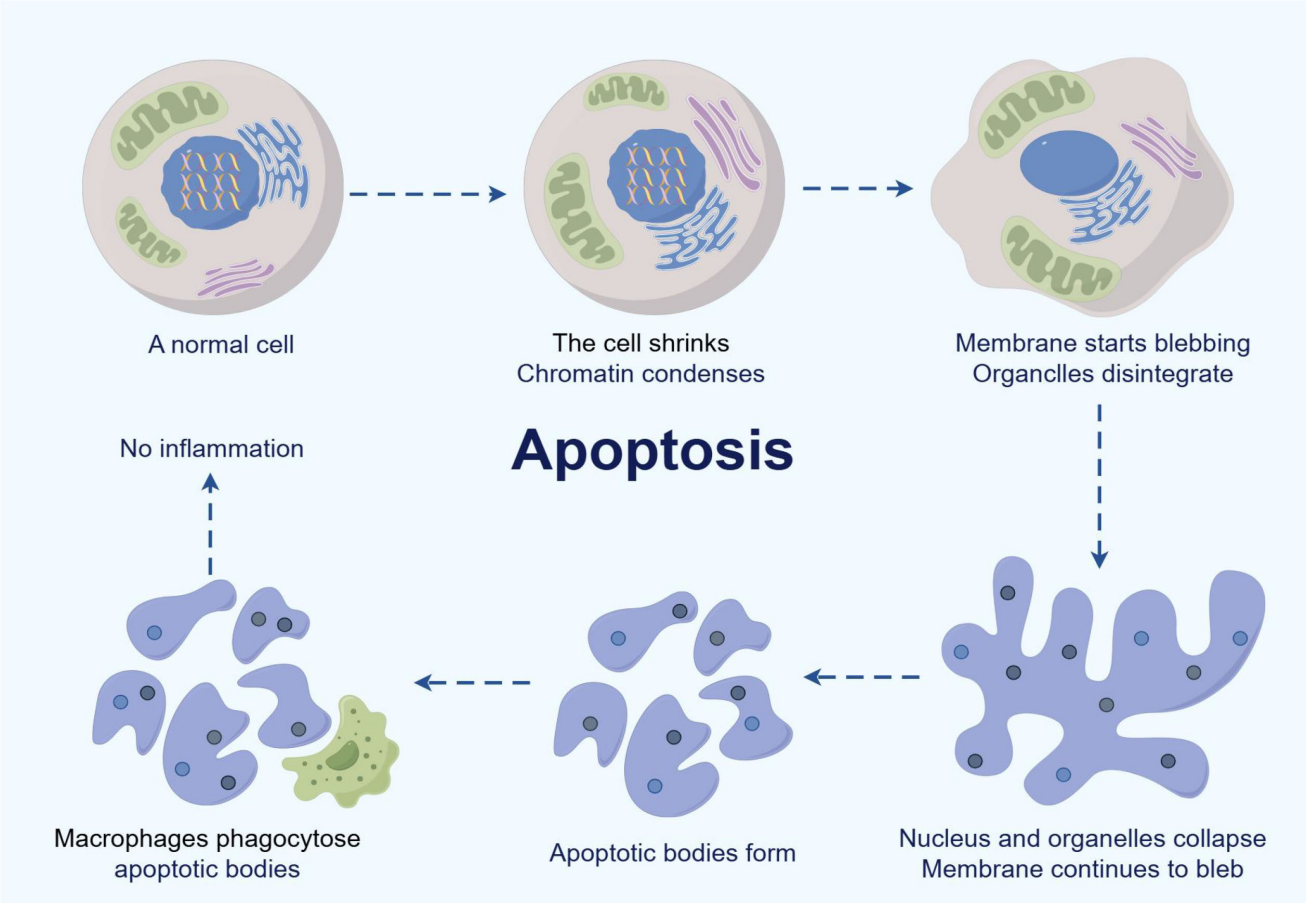
The process of apoptosis (by Figdraw). This figure contrasts a normal cell (left panel) with the sequential stages of apoptosis and subsequent phagocytosis by macrophages (right panel), highlighting the absence of inflammatory responses during programmed cell death.

Apoptosis is a complex, multi-step process that involves a cascade of tightly coordinated intracellular events. Morphologically, the apoptotic process is characterized by distinct changes, such as cell contraction, chromatin condensation, membrane blebbing, organelle disintegration, and the formation of apoptotic bodies. During the early stages, cells exhibit shrinkage and a reduction in volume, followed by the appearance of membrane protrusions—also known as blebs—which are hallmark features of apoptosis. Simultaneously, chromatin condenses and the nuclear fragments, signaling the irreversible commitment to cell death. As the process progresses, cells lose their structural and functional integrity, ultimately leading to the formation of apoptotic bodies—membrane-bound vesicles containing nuclear and cytoplasmic components. These apoptotic bodies are promptly recognized and engulfed by professional phagocytes such as macrophages. Importantly, this phagocytic clearance occurs without triggering an inflammatory response. In fact, macrophages actively secrete anti-inflammatory cytokines during the engulfment of apoptotic bodies, thereby contributing to the resolution of inflammation and promoting tissue repair and regeneration. Thus, apoptosis is not merely a destructive process but also plays a constructive role in maintaining tissue health and immune equilibrium. Through the coordinated execution of intracellular death signals and subsequent non-inflammatory clearance by immune cells, apoptosis serves as a key mechanism for preserving the internal environment and preventing pathological conditions. These processes are illustrated in **Figure 2**.

**Figure 3:**
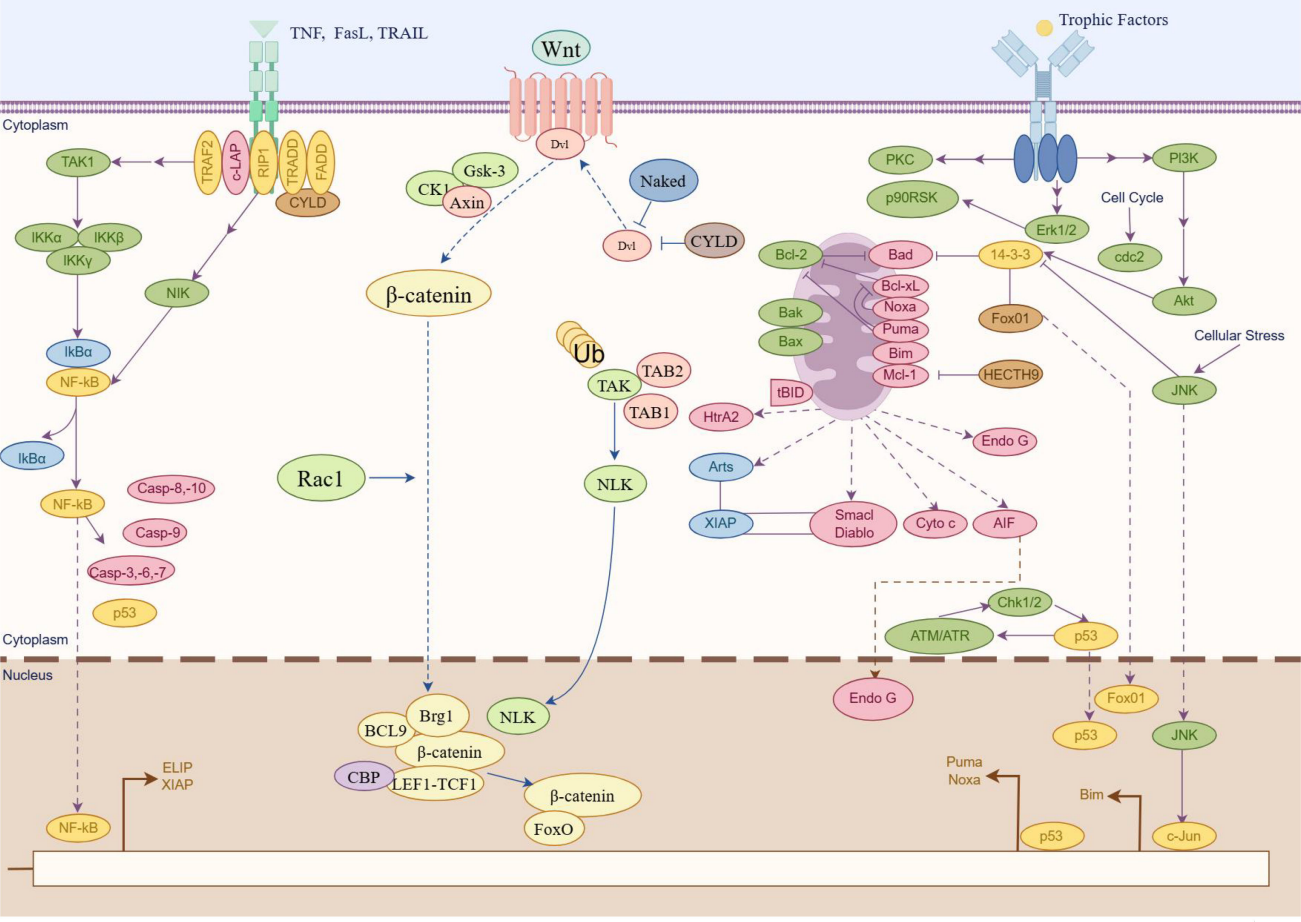
Major apoptosis signaling pathways participate in RA (by Figdraw). Schematic illustration of three pivotal signaling pathways involved in apoptosis regulation. The graphical representation delineates molecular cascades mediated by NF-κB signaling pathway, Wnt/β-catenin signaling pathway, and PI3K/Akt signaling pathway, highlighting their respective roles in cellular survival/death regulatory networks. Key molecular components are annotated to demonstrate pathway activation mechanisms and their functional convergence in apoptosis modulation.

Apoptosis is a common and essential form of programmed cell death that occurs through two main pathways: exogenous apoptotic pathways (death receptor–mediated) and endogenous apoptotic pathways (mitochondria-mediated). In the extrinsic apoptotic pathway, apoptosis is initiated when extracellular death ligands bind to their respective death receptors on the cell surface. This binding triggers the oligomerization of Fas-associated protein with death domain, which subsequently activates caspase-8 (51). Activated caspase-8 then initiates apoptosis through two mechanisms: directly cleaving and activating caspase-3, a key executioner caspase, and cleaving BH3-interacting domain death agonist (BID). The truncated BID then promotes the activation of proapoptotic Bcl-2 family members Bax and Bak, facilitating their insertion and oligomerization in the mitochondrial membrane. Simultaneously, activation of the extrinsic pathway also converges on the mitochondrial pathway, leading to the release of Cyt-c from mitochondria. Cyt-c interacts with Apaf-1 to form apoptosomes, which subsequently activate downstream caspase-9 and caspase-3, ultimately executing the apoptotic process. In addition to these pathways, the endoplasmic reticulum stress–mediated apoptotic pathway is increasingly recognized as a key mechanism of cell apoptosis. Recent research has highlighted that an imbalance between the proliferation and apoptosis of RA-FLS is closely associated with the progression of RA. Several signaling pathways, such as the Wnt/β-catenin, PI3K/AKT, and NF-κB signaling pathways, are implicated in the regulation of apoptosis and play an important role in RA (**Figure 3**). Therefore, elucidating the relationship between apoptosis and RA, especially through the perspective of ncRNA-mediated regulation, is essential.

### 3.2 Major apoptosis signaling pathways participate in RA

#### 3.2.1 NF-κB signaling pathway

The NF-κB signaling pathway plays a crucial role in the pathogenesis of RA, particularly in the regulation of apoptosis in synovial cells (52). The activation of NF-κB involves two major signaling pathways: the canonical (or classical) and non-canonical (or alternative) pathways (53). The canonical NF-κB pathway predominantly activates transcription factors such as p50, p65, and c-Rel, core members of the classical NF-κB family. It is rapidly and transiently triggered by various extracellular stimuli, including cytokine receptors, Tumor necrosis factor receptors (TNFRs), G protein-coupled receptors (GPCRs), T cell receptors (TCRs), and B cell receptors (BCRs). In contrast, the non-canonical pathway involves NF-κB–inducing kinase (also known as MAP3K14), inhibitor of nuclear factor kappa-B kinase subunit alpha (IKKα), and transcription factor RelB/p52 (RelB/p52) heterodimers (acting as transcription factors). Research has demonstrated a strong association between NF-κB activation and the inflammatory processes that characterize RA, leading to the insufficient apoptosis of synovial cells. For instance, IL-1β has been shown to promote apoptosis in synovial cells through the NF-κB pathway, underscoring its importance in RA pathophysiology (54). Recent research highlights the therapeutic potential of targeting NF-κB signaling with herbal formulations to modulate inflammation and apoptosis in RA. For example, Heilaohuacid G, a novel triterpenoid isolated from *Kadsura coccinea*, was shown to induce apoptosis and alleviate inflammation in RA-FLS through suppressing the NF-κB pathway (55). These findings suggest that modulation of the NF-κB pathway using TCM could be an effective strategy for slowing RA progression and improving clinical outcomes.

In summary, the NF-κB signaling pathway is a key mediator in the development of RA, particularly in regulating synovial cell apoptosis. Targeting this pathway through traditional herbal therapeutics offers a promising avenue for RA management.

#### 3.2.2 Wnt/β-catenin signaling pathway

Wnt proteins constitute a large family of secreted lipid-modified glycoproteins involved in key cellular processes such as differentiation, proliferation, apoptosis, and migration. The Wnt/β-catenin pathway—also known as the canonical Wnt pathway—comprises four segments: the extracellular signal, membrane segment, cytoplasmic segment, and nuclear segment. This pathway is activated when Wnt ligands bind to members of the Frizzled receptor family on the cell surface. A critical regulatory complex in this pathway involves β-catenin and Axin. A growing body of research indicates that the Wnt/β-catenin signaling pathway is closely involved in the regulation of apoptosis in RA. Liang et al. investigated the role of ZNRF3, a negative regulator of Wnt signaling, in RA-FLS apoptosis. Their study demonstrated that silencing ZNRF3 reduced apoptosis and enhanced cell viability (56, 57). Furthermore, ZNRF3 overexpression was shown to attenuate knee joint damage and decrease the level of inflammatory cytokine in a CIA mouse model. These effects may be attributed to the crosstalk between the Wnt and NF-κB pathways. In the context of TCM, several compounds have been found to promote apoptosis by modulating the Wnt/β-catenin signaling pathway. For example, 7-hydroxycoumarin has been reported to alleviate disease severity in CIA rats by inhibiting FLS proliferation and inducing apoptosis through the suppression of the Wnt/β-catenin signaling pathway (58).

Overall, the Wnt/β-catenin signaling pathway plays a crucial role in regulating apoptosis and the progression of RA. Targeting this pathway using TCM offers a promising avenue for the development of novel therapeutic strategies aimed at controlling RA pathogenesis.

#### 3.2.3 PI3K/Akt pathway

PI3Ks are a family of lipid kinases that phosphorylate phosphatidylinositols at the D3 position of the inositol ring. PI3K is typically activated by receptor tyrosine kinases, leading to the conversion of phosphatidylinositol (4,5)-bisphosphate into phosphatidylinositol (3,4,5)-trisphosphate. This event subsequently promotes the phosphorylation and activation of Akt, a central downstream effector of the PI3K pathway. Once activated, Akt translocates from the plasma membrane to the cytoplasm and nucleus, where it phosphorylates a variety of substrates that regulate crucial cellular processes, including apoptosis (59). Recent studies have highlighted the relevance of the PI3K/AKT pathway in the regulation of apoptosis in RA. For instance, upregulation of miR-365 has been shown to induce apoptosis and inhibit proliferation of RA-FLS through the downregulation of IGF1 and the inhibition of the PI3K/AKT/mechanistic target of rapamycin (mTOR) pathway (60). Abnormal activation of this pathway has been strongly associated with RA progression. In the realm of TCM, several compounds have demonstrated the ability to promote RA cell apoptosis by regulating the PI3K/AKT signaling pathway, thereby exerting their anti-inflammatory effects. For example, baicalin, a bioactive flavonoid derived from *Scutellaria baicalensis*, has been shown to inhibit the PI3K/AKT signaling pathway, induce apoptosis in RA cells, and reduce inflammatory responses (61). Regulating the PI3K/AKT signaling pathway through TCM to promote apoptosis in RA cells may offer novel insights and therapeutic strategies for the treatment of RA.

To sum up, the PI3K/AKT signaling pathway plays a key role in the regulation of apoptosis in RA. Modulation of this pathway through TCM offers a promising therapeutic approach for controlling RA progression and improving clinical outcomes.

## 4 Mechanism of apoptosis regulated by ceRNA in RA

The ceRNA network involves complex regulatory interactions among lncRNAs, miRNAs, and mRNAs, which collectively influence gene expression and modulate various cellular processes, including apoptosis. In RA, dysregulation of apoptosis can lead to synovial hyperplasia and joint destruction, thereby promoting disease progression. A brief summary of key findings is provided in **Table 2**.

**Table 2:** Mechanism of apoptosis regulated by ceRNA in RA.

ncRNAs	Target miRNAs	Related genes	Dysregulation	Cell models	References
lncRNAs					
lncRNA AL928768.3	/	NF-κB pathway	Upregulation	RA-FLS	(62)
lncRNA THRIL	/	PI3K/AKT pathway	Upregulation	RA-FLS	(63)
LINC00152	/	FOXM1/Wnt/β-catenin pathway	Upregulation	RA-FLS	(64)
lncRNA HOTTIP	/	TLR4	Upregulation	RA mouse model/RA-FLS	(65)
lncRNA GAS5	miR-361-5p	PDK4	Upregulation	RA-FLS	(74)
lncRNA ZFAS1	miR-3926	FSTL1	Upregulation	RA-FLS	(66)
lncRNA ZFAS1	miR-2682-5p	ADAMTS9	Upregulation	RA-FLS	(67)
lncRNA ZFAS1	miR-296-5p	MMP-15	Upregulation	RA mouse model/MH7A	(68)
lncRNA XIST	miR-126-3p	NF-κB pathway	Upregulation	RA-FLS	(69)
lncRNA XIST	miR-34a-5p	YY1	Upregulation	AIA rats	(70)
lncRNA NEAT1	miR-410-3p	YY1	Upregulation	RA-FLS	(75)
lncRNA PVT1	miR-145-5p	/	Upregulation	RA-FLS	(71)
lncRNA PVT1	/	Sirt6	Upregulation	RA-FLS	(72)
lncRNA PVT1	miR-543	SCUBE2	Upregulation	synovial tissues of RA rats	(73)
lncRNA MAPKAPK5-AS1	miR-146a-3p	SIRT1/NF-κB	Downregulation	RA-PBMCs/RA-FLS	(76)
lncRNA HOTAIR	miR-106b-5p	/	Downregulation	RA-FLS	(77)
lncRNA OSER1-AS1	miR-1298-5p	E2F1	Downregulation	RA-FLS	(78)
lncRNA TSPEAR	miR-212-3p	/	Downregulation	RA-FLS	(79)
lncRNA CAIF	miR-20a	/	Downregulation	RA-FLS	(80)
lncRNA BZRAP1-AS1	miR-1286	COL5A2	Downregulation	RA-FLS	(81)
lncRNA GAS5	miR-128-3p	HDAC4	Downregulation	RA-FLS	(82)
lncRNAGAS5	miR-222-3p	SIRT1/NF-κB	Downregulation	RA-FLS	(83)
lncRNA CASC2	/	IL-17	Downregulation	RA-FLS	(84)
lncRNA DILC	/	IL-6	Downregulation	RA-FLS	(85)
circRNAs					
circ_0088036	miR-1263	REL/NF-κB	Upregulation	RA-FLS	(86)
circ_0088036	miR-326	FZD4	Upregulation	RA-FLS	(87)
circ_0002984	miR-543	PCSK6	Upregulation	RA-FLS	(88)
circ_0000479	miR-766	FKBP5	Upregulation	RA-FLS	(89)
circ_0083964	miR-204-5p	YY1	Upregulation	RA-FLS	(90)
circ_0004712	miR-633	TRAF6	Upregulation	RA-FLS	(91)
circPTTG1IP	miR-431-5p	FSTL1	Upregulation	RA-FLS	(92)
circ_0088194	miR-30a-3p	ADAM10	Upregulation	RA-FLS	(93)
circ_0025908	miR-650	SCUBE2	Upregulation	RA-FLS	(94)
circ_0001947	miR-671-5p	STAT3	Upregulation	RA-FLS	(95)
circ_0025908	miR-137	HIPK2	Upregulation	RA-FLS	(96)
circ_0003972	miR-654-5p	FZD4	Upregulation	RA-FLS	(97)
circMAPK9	miR-140-3p	PPM1	Upregulation	RA-FLS	(98)
circASH2L	miR-129-5p	HIPK2	Upregulation	RA-FLS	(99)
circ-AFF2	miR-650	CNP	Upregulation	RA-FLS	(100)
circ_0000396	miR-574-5p	RSPO1	Downregulation	RA-FLS	(101)
circ_0008360	miR-135b-5p	HDAC4	Downregulation	RA-FLS	(102)
circ-Sirt1	miR-132	Sirt1	Downregulation	RA-FLS	(103)

### 4.1 Upregulated lncRNAs

#### 4.1.1 lncRNAs associated with apoptotic pathways

lncRNAs play a significant role in the pathogenesis of RA through the regulation of the apoptosis-related signaling pathway. lncRNA AL928768.3 is significantly overexpressed in RA-FLS and has been shown to promote proliferation, invasion, and inflammation while inhibiting apoptosis. This effect is mediated by activating lymphotoxin beta–mediated NF-κB signaling (62). TNF and heterogeneous nuclear RNPL (hnRNPL)–related immunoregulatory lncRNA (THRIL) has been identified as another key regulatory lncRNA in RA. THRIL forms a complex with hnRNPL in the nucleoplasm. Liang et al. revealed that THRIL could regulate apoptosis and the inflammatory response in RA-FLS by activating the PI3K/AKT signaling pathway, thereby contributing to RA pathogenesis (63). LINC00152 presented the highest fold change in expression in RA-FLS according to microarray analysis (64). Further investigation revealed that LINC00152 functioned as a molecular sponge for miR-1270, thereby upregulating FOXM1 expression. This cascade promoted proliferation and induced apoptosis by activating the Wnt/β-catenin pathway. HOXA distal transcript antisense RNA (HOTTIP), located near chromosome 7p15.2, is another lncRNA elevated in synovial tissues of the RA mouse model. Mechanistically, HOTTIP recruits MLL1 to induce methylation of the TLR4 promoter, leading to suppression of RA-FLS proliferation and promotion of apoptosis and inflammatory responses (65). These findings suggest that HOTTIP plays a crucial regulatory role in RA progression by modulating the TLR4 signaling pathway and influencing FLS apoptosis and inflammation.

#### 4.1.2 lncRNA ZFAS1

lncRNA ZFAS1, located on chromosome 20q13, has been found to be aberrantly expressed in various human diseases. Knockdown of lncRNA ZFAS1 in RA-FLSs has been shown to suppress cell proliferation, migration, and invasion, reduce inflammatory cytokine production, and induce apoptosis (66). Mechanistically, lncRNA ZFAS1 functions as a ceRNA by sponging miR-3926, thereby regulating FSTL1 expression. In another study, Yang et al. reported that lncRNA ZFAS1 regulated the proliferation, apoptosis, inflammatory response, and autophagy of RA-FLS via the miR-2682-5p/ADAMTS9 axis, suggesting its potential as a potential therapeutic target for RA (67). Similarly, lncRNA ZFAS1 has been identified as a ceRNA for miR-296-5p (68). Its overexpression correlates with the downregulation of miR-296-5p and the upregulation of MMP-15, ultimately promoting proliferation and suppressing apoptosis in MH7A cells. In summary, lncRNA ZFAS1 plays a crucial role in the pathogenesis of RA by functioning as a ceRNA. These findings provide valuable insights into its therapeutic potential, and further research is warranted to elucidate the precise mechanisms and regulatory networks involving lncRNA ZFAS1 in RA.

#### 4.1.3 lncRNA XIST

lncRNA XIST gene, located on chromosome Xq13.2, has been implicated in the regulation of apoptosis in RA. Liu et al. observed that lncRNA XIST is significantly upregulated in both synovial tissues and RA-FLSs (69). Overexpression of lncRNA XIST was found to promote cell proliferation and inhibit apoptosis, primarily through its function as a ceRNA that sponges miR-126-3p, thereby activating the NF-κB pathway. Additionally, in an animal model, Wei et al. demonstrated that lncRNA XIST promoted the expression of YY1 by competitively binding to miR-34a-5p, which subsequently promoted cell migration and proliferation, as well as inhibited apoptosis (70). In summary, lncRNA XIST contributes to RA pathogenesis by modulating apoptosis-related signaling through ceRNA mechanisms. These findings suggest that targeting lncRNA XIST may offer promising therapeutic opportunities for RA treatment.

#### 4.1.4 lncRNA PVT1

The plasmacytoma variant translocation 1 (PVT1) gene, located on chromosome band 8q24.21, has been implicated in the pathogenesis of RA. Tang et al. have reported that lncRNA PVT1 is substantially upregulated in synovial tissues and RA-FLSs (71). Mechanistically, lncRNA PVT1 can regulate apoptosis and inflammatory responses by targeting miR-145-5p and activating the NF-κB pathway. In another study, the knockdown of lncRNA PVT1 was shown to suppress RA-FLS proliferation and inflammation while inducing apoptosis by inhibiting sirt6 methylation, thus contributing to RA progression attenuation (72). Furthermore, lncRNA PVT1 silencing was found to inhibit pro-inflammatory cytokine secretion and inhibit apoptosis in RA-FLSs, with the effect being mediated via the lncRNA PVT1/miR-543/SCUBE2 axis (73). These findings underscore the critical regulatory role of lncRNA PVT1 in modulating RA-FLS apoptosis and inflammation, suggesting its potential as a therapeutic target in RA management.

#### 4.1.5 lncRNA GAS5

The lncRNA *growth arrest-specific transcript 5* (GAS5) gene is located at chromosome 1q25 and has been implicated in RA pathophysiology. Zhang et al. investigated the molecular mechanisms underlying the lncRNA GAS5/miR-361-5p/PDK4 regulatory axis in RA (74). Given the risk of joint damage and disability associated with RA, understanding apoptosis-related regulatory networks is crucial for the development of novel therapeutic strategies. Using a dual-luciferase reporter assay, the study confirmed direct binding interactions between lncRNA GAS5, miR-361-5p, and PDK4. The results revealed that upregulation of both lncRNA GAS5 and PDK4 in RA-FLSs enhanced cell proliferation and inhibited apoptosis. In summary, this study highlights the role of the lncRNA GAS5/miR-361-5p/PDK4 axis in the suppression of apoptosis in RA. Targeting lncRNA GAS5 may provide a promising therapeutic strategy to restore apoptosis balance and control RA progression. These insights enhance our understanding of the molecular mechanisms underlying insufficient apoptosis in RA and may guide future treatment development.

#### 4.1.6 lncRNA NEAT1

lncRNA *nuclear paraspeckle assembly transcript 1* (NEAT1), located on chromosome 11q13.1, has been implicated in the regulation of apoptosis in RA. Wang et al. investigated the role of NEAT1 in RA-FLSs (75) and found that it was significantly upregulated in RA synovial tissues and RA-FLSs. Upregulation of NEAT1 promoted cell proliferation and suppressed apoptosis in RA-FLSs. Mechanistically, NEAT1 exerts its effects through the miR-410-3p/YY1 axis. The study demonstrated that NEAT1 directly interacted with miR-410-3p, acting as a molecular sponge, thereby downregulating miR-410-3p and, consequently, upregulating its downstream target, YY1. This regulatory cascade disrupts apoptosis regulation in RA-FLSs and highlights the importance of NEAT1 in the pathophysiology of RA. Targeting NEAT1 and its associated pathways may offer innovative therapeutic strategies for RA, potentially leading to more effective treatment options. Overall, lncRNA NEAT1 represents a promising therapeutic target for modulating apoptosis and fibrosis in RA.

### 4.2 Downregulated lncRNAs

In the pathogenesis of RA, lncRNAs play a crucial role in regulating apoptosis. Specifically, m6A-modified lncRNA MAPKAPK5-AS1 has been shown to influence RA progression by modulating the miR-146a-3p/SIRT1/NF-κB axis (76). This study highlights a novel lncRNA/miRNA/mRNA regulatory axis and an m6A-dependent mechanism involved in RA-associated apoptosis, providing potential diagnostic and therapeutic strategies. Additionally, lncRNA HOTAIR has demonstrated a protective role in RA by promoting apoptosis through the miR-106b-5p/Smad7 axis (77). Overexpression of HOTAIR can significantly enhance RA-FLS apoptosis, suggesting a mechanism of action similar to that of MAPKAPK5-AS1. Another downregulated lncRNA, lncRNA OSER1-AS1, has also been identified as a key player in RA (78). It regulates inflammation and apoptosis via the miR-1298-5p/E2F1 axis. Moreover, lncRNA TSPEAR-AS2 is reported to be downregulated in RA, and its overexpression decreases RA-FLS apoptosis by downregulating miR-212-3p expression (79). Similarly, lncRNA cardiac arrhythmia inhibition factor (CAIF) may suppress RA-FLS apoptosis by promoting the maturation of miR-20a (80). Through these mechanisms, lncRNAs may restore the balance between pro- and anti-apoptotic signals, helping to alleviate RA symptoms and slow disease progression.

Recent research has also highlighted the importance of the lncRNA BZRAP1-AS1/miR-1286/COL5A2 axis in apoptosis regulation during RA progression (81). The interaction between lncRNAs and miRNAs constitutes a key component of the gene regulatory networks controlling RA cell fate. For instance, lncRNA GAS5 has been shown to alleviate RA development by regulating the miR-128-3p/HDAC4 signaling pathway (82), whereas its modulation of the miR-222-3p/Sirt1 axis represents a novel approach for managing RA (83). These findings emphasize the complexity and therapeutic potential of ncRNA-mediated regulation in autoimmune disorders. Furthermore, several studies have shown that downregulation of lncRNA CASC2 is observed in RA and that its overexpression may promote RA-FLS apoptosis by downregulating IL-17 (84). Similarly, lncRNA DILC may contribute to RA pathogenesis by inducing apoptosis of RA-FLS and downregulating IL-6 (85).

**Figure 4:**
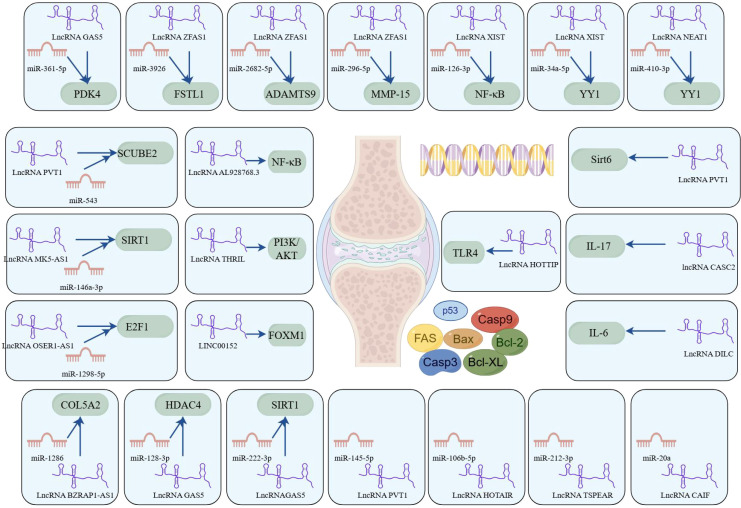
LncRNAs play a ceRNA regulatory role in RA (by Figdraw). This figure delineates the intricate interplay between lncRNAs, miRNAs, and their downstream target genes or signaling pathways, with a focus on apoptosis regulation. These networks were curated from experimental and bioinformatic analyses, underscoring lncRNAs as central regulators of apoptosis-associated pathways. The diagram highlights potential therapeutic targets for RA, where dysregulated ncRNA interactions contribute to pathogenic mechanisms.

In summary, lncRNA plays a crucial role in the pathogenesis of RA, particularly in regulating apoptosis, as illustrated in **Figure 4**. Continued research into these lncRNAs and their interactions with miRNAs and downstream effectors may yield novel therapeutic targets and strategies for RA management.

### 4.3 Upregulated circRNAs

The networks of circRNA-miRNA-mRNA play a critical role in the pathogenesis of RA, especially by modulating apoptosis and inflammation through key signaling pathways such as the NF-κB pathway. circ_0088036 has been shown to activate the NF-κB pathway through the miR-1263/REL axis, thereby promoting inflammatory responses of RA-FLS and inhibiting apoptosis (86). Another study supported similar findings, demonstrating that circ_0088036 facilitates RA-FLS proliferation and inflammation while inhibiting apoptosis through the miR-326/FZD4 axis (87). Moreover, circ_0002984 has been identified as a key regulator in RA by modulating the miR-543/PCSK6 signaling axis (88). This circRNA is upregulated in RA-FLSs and proliferation, migration, and inflammatory cytokine secretion while inhibiting apoptosis. The underlying mechanism involves circ_0002984 sponging miR-543, leading to increased expression of PCSK6. Notably, knocking down circ_0002984 reverses these pathological processes, highlighting its potential as a therapeutic target in RA. Similarly, circ_0000479 was found to promote RA-FLS proliferation, invasion, migration, and inflammatory responses while inhibiting apoptosis via the miR-766/FKBP5 axis (89). This pathway emphasizes the multifaceted role of circ_0000479in RA progression and underscores its function as a critical regulatory node in the apoptotic imbalance observed in RA.

Recent studies have increasingly highlighted the significant role of circRNAs in the regulation of apoptosis in RA-FLS, especially through their function as ceRNAs. For instance, circ_0083964 has been shown to suppress proliferation, metastasis, and inflammation while facilitating apoptosis in RA-FLSs by targeting the miR-204-5p/YY1 axis (90). The knockdown of circ_0083964—via miR-204-5p–mediated YY1—may provide a theoretical basis for the treatment of RA. Similarly, circ_0004712 was found to modulate apoptosis in RA-FLSs by sponging miR-633 and thereby regulating TRAF6 expression (91). These findings illustrate the intricate interplay between circRNAs and miRNAs in modulating cellular functions and suggest that targeting such regulatory networks may provide new avenues for therapeutic intervention in RA. In addition, circPTTG1IP has been reported to facilitate apoptosis through positively regulating FSTL1 expression by sponging miR-431-5p in RA-FLSs (92). Another circRNA, circ_0088194, promotes proliferation, migration, and inflammatory response while inhibiting apoptosis in RA-FLSs by acting on the miR-30a-3p/ADAM10 axis (93). Modulating this pathway could offer an effective approach to inhibit the progression of RA and present a new strategy for clinical treatment.

Furthermore, circ_0025908 has been shown to play a critical role in modulating cellular behaviors, such as proliferation, migration, invasion, and inflammation (94). Specifically, the knockdown of circ_0025908 leads to a marked inhibition of these processes through the regulation of miR-650-dependent SCUBE2, ultimately stimulating apoptosis in FLS. The regulatory role of circRNAs in apoptosis is further underscored by the study of circ_0001947, whose expression is significantly altered in RA patients (95). This circRNA enhances STAT3 expression by sponging miR-671-5p, thereby influencing FLS proliferation, migration, invasion, and apoptosis. Moreover, circ_0025908 also inhibits apoptosis in RA by targeting the miR-137/HIPK2 axis, providing us with new therapeutic targets and strategies (96). Beyond these specific examples, numerous ceRNA regulatory networks have been implicated in RA-associated apoptosis, including circ_0003972/miR-654-5p/FZD4 axis, circMAPK9/miR-140-3p/PPM1A axis, circASH2L/miR-129-5p/HIPK2 axis, and circ-AFF2/miR-650/CNP axis (97–100). Overall, accumulating evidence supports the crucial role of circRNA-mediated ceRNA networks in regulating apoptosis of RA-FLSs. These findings not only enhance our understanding of RA pathogenesis but also offer promising molecular targets for future therapeutic development.

### 4.4 Downregulated circRNAs

**Figure 5:**
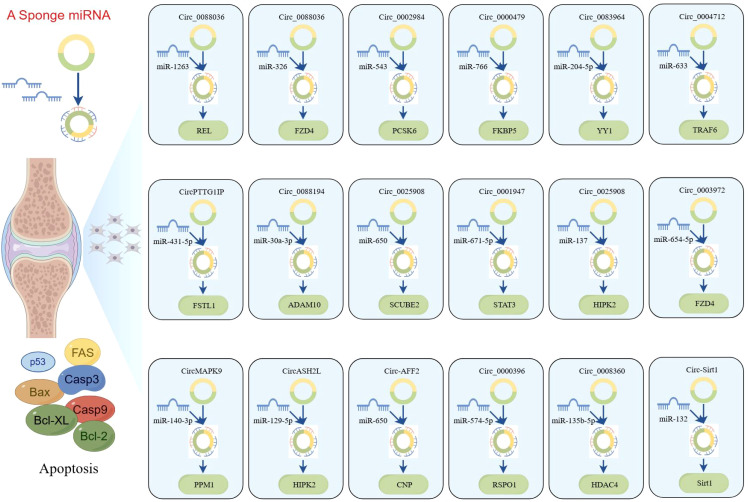
CircRNAs play a ceRNA regulatory role in RA (by Figdraw). This figure illustrates the ceRNA networks mediated by circRNAs, which sequester miRNAs to modulate the expression of downstream target genes involved in apoptosis. The diagram further integrates core apoptotic regulators, including Fas, Bax, caspase-3/9, and Bcl-2 family proteins, highlighting how circRNA-miRNA crosstalk fine-tunes cell fate decisions. These networks were constructed on the basis of RNA-seq, miRNA pull-down assays, and functional validations, underscoring circRNAs as critical nodes in apoptosis regulation. Dysregulation of these axes may contribute to diseases characterized by aberrant cell survival of RA.

Compared to healthy controls, circ_0000396 is notably downregulated in the synovial tissues of RA patients. Functionally, circ_0000396 acts as a molecular sponge for miR-574-5p, thereby decreasing its expression and reversing the suppression of RSPO1. This axis promotes RA-FLS proliferation and inflammation while inhibiting apoptosis (101). The intricate interaction between circ_0000396 and miR-574-5p underscores their pivotal roles in regulating apoptotic signaling and inflammatory responses in RA. These findings not only enhance our understanding of the molecular mechanisms underlying RA but also open avenues for potential therapeutic interventions targeting these circRNAs. Another downregulated circRNA, circ_0008360, has been shown to sponge miR-135b-5p, leading to upregulation of HDAC4. This interaction inhibits proliferation, migration, and inflammation while facilitating apoptosis in RA-FLSs (102). Similarly, circ-Sirt1 is downregulated in RA, and its loss leads to the upregulation of miR-132, which suppresses Sirt1 signaling. Restoration of circ-Sirt1 levels can inhibit RA-FLS proliferation and induce apoptosis through activation of the Sirt1 pathway (103). A better understanding of these molecular mechanisms will facilitate the development of novel therapeutic approaches for the prevention and treatment of RA, as illustrated in **Figure 5**.

## 5 TCM treats RA by regulating ncRNAs

TCM has a long-standing history and demonstrated efficacy in preventing and treating RA. TCM exerts therapeutic effects through multiple mechanisms, including the modulation of inflammatory responses, inhibition of apoptosis, mitigation of oxidative stress, and improvement of hypercoagulability. In recent years, considerable progress has been made in understanding how TCM promotes apoptosis in RA via the regulation of ceRNA networks. This highlights the potential of a multidisciplinary approach to explore the interactions between lncRNAs, circRNAs, and traditional herbal treatments in the management of RA, as summarized in **Table 3**.

**Table 3:** Traditional Chinese medicine treats RA by regulating ncRNAs.

TCM	ncRNAs	Expression	Cell model	Influence on apoptosis	References
Brucine	circ_0139658/miR-653-5p/YY1	Downregulation	RA-FLS	Promoting	(115)
Triptolide	lncRNA RP11-83J16.1/URI1/β-catenin	Downregulation	RA-FLS	Promoting	(106)
Triptolide	lncRNA ENST00000619282	Downregulation	RA-FLS	Promoting	(107)
Tanshinone IIA	lncRNA GAS5	Upregulation	RA-FLS	Promoting	(109)
Quercetin	lncRNA MALAT1	Upregulation	RA-FLS	Promoting	(111)
Paeoniflorin	lncRNA MALAT1/Wnt1/β-catenin	Upregulation	RA-FLS	Promoting	(113)
Xinfeng Capsule	lncRNA MAPKAPK5-AS1	Upregulation	RA-PBMCs/RA-FLS	Promoting	(118)
Xinfeng Capsule	circ-CBLB	Upregulation	RA-FLS	Promoting	(122)

### 5.1 Triptolide

Triptolide is a major bioactive diterpenoid triepoxide compound extracted from *Tripterygium wilfordii* Hook. F., a classic TCM remedy that has long been used in China for its anti-rheumatic properties (104, 105). Accumulating evidence suggests that triptolide can promote RA-FLS apoptosis and decrease proliferation, invasion, and inflammation through inactivating lncRNA RP11-83J16.1–mediated URI1 and β-catenin signaling (106). Similarly, another study by Wen et al. found that triptolide downregulated lncRNA ENST00000619282, thereby promoting apoptosis and reducing inflammation in RA (107). While these findings underscore the potential of triptolide in modulating lncRNA expression to treat RA, the precise mechanisms underlying these regulatory pathways remain to be fully explored. Future studies should focus on dissecting these pathways and clarify the specific interactions between triptolide and ncRNAs, with the goal of optimizing its clinical application in RA therapy.

### 5.2 Tanshinone IIA

A key therapeutic strategy for RA is the induction of apoptosis in RA-FLS. Tanshinone IIA, a bioactive compound extracted from Danshen, possesses well-documented anti-inflammatory, pro-apoptotic, and antioxidant properties. It has been shown to exert protective effects on cardiomyocytes and delay tumor progression (108). In the context of RA, Tanshinone IIA has demonstrated therapeutic potential through promoting RA-FLS apoptosis by upregulating lncRNA GAS5. This upregulation enhances the expression of caspase3/caspase9 while simultaneously inhibiting the PI3K/AKT signaling (109). Although lncRNA GAS5 has emerged as a promising target in the treatment of RA, the precise molecular mechanisms by which Tanshinone IIA regulates lncRNA GAS5 expression and downstream apoptotic pathways remain to be further studied.

### 5.3 Quercetin

Quercetin is a naturally occurring polyphenolic flavonoid widely found in edible plants. It exhibits multiple biological activities, such as anti-inflammatory, anti-oxidation, anti-proliferative, and pro-apoptotic properties (110). One important lncRNA implicated in various diseases is metastasis-associated lung adenocarcinoma transcript 1 (MALAT1), an extensively studied transcript over 8,000 nucleotides in length, located on chromosome 11q13. A study by Pan et al. demonstrated that quercetin can promote RAF-LS apoptosis by upregulating lncRNA MALAT1 and activating the PI3K/AKT pathway (111). This finding underscores the potential of lncRNA MALAT1 as a critical regulator in quercetin-induced apoptosis. However, the interplay between quercetin, MALAT1, and the PI3K/AKT pathway appears to involve a complex regulatory mechanism that warrants further investigation to fully elucidate the underlying molecular processes.

### 5.4 Paeoniflorin

Paeoniflorin (PF) is a monoterpene glucoside and the primary bioactive component extracted from the roots of *Paeonia* plants. It has been widely recognized for its anti-inflammatory and pro-apoptotic effects in various inflammatory disorders (112). The Wnt1/β-catenin signaling is known to play a critical role in regulating apoptosis and cellular injury. Recent research has shown that PF promotes RA-FLS apoptosis by upregulating the expression of lncRNA MALAT1 and inhibiting the Wnt1/β-catenin pathway (113). This finding highlights a novel mechanism and therapeutic target for RA treatment, offering a promising strategy for further investigation and clinical application.

### 5.5 Brucine

Brucine is an indole alkaloid extracted from the seeds of *Strychnos nux-vomica* L. (Loganiaceae), known for its diverse pharmacological activities. It has recently attracted attention for its protective effects in RA (114). A latest study demonstrated that brucine promoted apoptosis and suppressed proliferation, migration, invasion, and inflammation in RA-FLSs by decreasing YY1 via the circ_0139658/miR-653-5p axis (115). This regulatory mechanism further underscores the therapeutic potential of brucine in RA and provides a theoretical basis for its future clinical development and application.

### 5.6 Xinfeng capsule

XFC (Anhui Pharmaceutical Production Number: Z20050062, Patent Number: ZL 2013 1 0011369.8) is a TCM formula composed of *Astragalus membranaceus*, *Coicis semen*, *Tripterygium wilfordii*, and centipede. It has been clinically applied in the treatment of rheumatic diseases at the First Affiliated Hospital of Anhui University of Traditional Chinese Medicine for more than two decades. The formulation adheres to strict quality control standards, as confirmed by high-performance liquid chromatography fingerprinting (116). High-level clinical evidence supports the efficacy of XFC in RA treatment. Large-sample, multi-center randomized controlled trials, as well as a cohort study involving 10,000 patients, provide robust evidence-based validation for its therapeutic role in RA management (117, 118). Experimental studies further suggest that XFC can promote apoptosis *in vitro* and *in vivo* (119, 120). Mechanistically, XFC has been shown to promote apoptosis and inhibit inflammatory response in RA by upregulating lncRNA MAPKAPK5-AS1 (121). More recently, Li et al. showed that XFC exerted its anti-rheumatic effects by upregulating circ-CBLB, thereby promoting apoptosis and inhibiting proliferation and inflammatory responses in RA-FLSs (122). In addition to its anti-inflammatory and pro-apoptotic actions, XFC contains flavonoid-rich components that may provide both preventive and therapeutic benefits in RA, aligning with the holistic principles of TCM focused on disease prevention and systemic balance.

## 6 Conclusions and future perspectives

In recent years, the incidence of RA has been on the rise, a trend attributed to the interplay of multiple complex factors, including genetic predisposition and biological mechanisms. Among emerging areas of research, lncRNAs and circRNAs—two novel classes of ncRNAs—have garnered increasing attention because of their pivotal roles in the pathogenesis of multiple diseases, particularly in the regulation of apoptosis in RA. This review focused on the roles of lncRNAs/circRNAs as potential biomarkers for the diagnosis of RA, with a particular emphasis on their function in apoptosis through ceRNA regulatory networks. Additionally, we summarized the current understanding of how TCM exerts therapeutic effects in RA by modulating ncRNA expression and ceRNA pathways. Despite accumulating evidence on the involvement of ncRNAs in RA, the potential feasibility of ncRNAs as RA therapy vectors remains largely unexplored. Many of these findings are still in the early stages of research, with limited validation in *in vitro*, *in vivo*, or clinical settings. Although certain ncRNAs have been identified as aberrantly expressed in RA, the functional implications and therapeutic relevance of numerous others remain unclear due to a lack of experimental investigation.

Moreover, differentially expressed ncRNAs identified in peripheral blood, joint tissues, or RA-FLSs have not yet been validated as reliable diagnostic or therapeutic biomarkers. One of the major challenges is determining whether these ncRNA expression patterns are unique to RA or are shared across other inflammatory or autoimmune diseases. Therefore, although specific ncRNAs have been identified in RA, their therapeutic potential may be limited by additional layers of regulation, such as albumin modification and methylation, which also contribute to RA pathogenesis. A comprehensive understanding of these complex interactions is crucial for developing effective ncRNA-based therapies for RA.

Furthermore, the ceRNA mechanism represents a primary mode of action through which ncRNAs exert their regulatory functions, suggesting that ncRNAs could serve as promising biomarkers or therapeutic targets for RA. Nevertheless, the specific mechanisms by which ceRNA networks regulate apoptotic pathways require further investigation. Programmed cell death (PCD) encompasses various mechanisms, including apoptosis, autophagy, pyroptosis, ferroptosis, and cuproptosis. The crosstalk between these different PCD modalities and ceRNA networks forms a highly intricate regulatory system. In the context of RA, the precise contributions of these interactions remain insufficiently understood and warrant further in-depth exploration.

In the diagnosis of RA, anti-citrullin peptide (anti-CCP) antibodies and rheumatoid factor (RF) are commonly used serological markers. However, reliance on these markers alone may not yield optimal diagnostic accuracy. Integrating ncRNAs with traditional serological markers such as RF and anti-CCP antibody may significantly improve the diagnostic accuracy of RA, providing a more reliable tool for clinical diagnosis and early detection of RA.

Although TCM has shown therapeutic potential in RA treatment, it also faces certain limitations, including low bioavailability, inconsistent efficacy, and potential side effects. In recent years, the application of nanotechnology has opened new avenues for the modernization and precise treatment of TCM. By combining the bioactive components of TCM with nanomaterials, solubility and bioavailability can be markedly improved, thereby enhancing therapeutic efficacy. Nanocarriers can be specifically engineered to enable targeted drug delivery, which minimizes off-target distribution, reduces side effects, and enhances treatment accuracy. Furthermore, the combination of nanotechnology and ncRNAs presents an innovative strategy to improve TCM-based therapies for RA. By modulating gene expression at the molecular level, nanotechnology can facilitate the delivery of ncRNA-targeting agents, amplifying the therapeutic benefits of TCM. Future research should continue to explore this synergistic approach to optimize the safety, specificity, and effectiveness of TCM in RA management.

In conclusion, the exploration of ncRNA-mediated apoptotic pathways offers promising opportunities for developing novel therapeutic strategies for RA. Targeting specific ncRNAs or their interactions with apoptotic regulators may provide a means to modulate cell death in RA, potentially alleviating clinical symptoms and improving patient outcomes. Continued investigation is necessary to fully elucidate these mechanisms and advance their translation into practical clinical applications.
